# Transglutaminase 2 Expression Is Increased as a Function of Malignancy Grade and Negatively Regulates Cell Growth in Meningioma

**DOI:** 10.1371/journal.pone.0108228

**Published:** 2014-09-23

**Authors:** Yin-Cheng Huang, Kuo-Chen Wei, Chen-Nen Chang, Pin-Yuan Chen, Peng-Wei Hsu, Carl P. Chen, Chin-Song Lu, Hung-Li Wang, David H. Gutmann, Tu-Hsueh Yeh

**Affiliations:** 1 Department of Neurosurgery, Chang Gung Memorial Hospital at Linkou, Taoyuan, Taiwan; 2 Department of Neurology, Chang Gung Memorial Hospital at Linkou, Taoyuan, Taiwan; 3 Neuroscience Research Center, Chang Gung Memorial Hospital at Linkou, Taoyuan, Taiwan; 4 Department of Rehabilitation, Chang Gung Memorial Hospital at Linkou, Taoyuan, Taiwan; 5 Chang Gung University, College of Medicine, Taoyuan, Taiwan; 6 Department of Neurology, Washington University, School of Medicine, St. Louis, Missouri, United States of America; University of Southern California, United States of America

## Abstract

Most meningiomas are benign, but some clinical-aggressive tumors exhibit brain invasion and cannot be resected without significant complications. To identify molecular markers for these clinically-aggressive meningiomas, we performed microarray analyses on 24 primary cultures from 21 meningiomas and 3 arachnoid membranes. Using this approach, increased transglutaminase 2 (TGM2) expression was observed, which was subsequently validated in an independent set of 82 meningiomas by immunohistochemistry. Importantly, the TGM2 expression level was associated with increasing WHO malignancy grade as well as meningioma recurrence. Inhibition of TGM2 function by siRNA or cystamine induced meningioma cell death, which was associated with reduced AKT phosphorylation and caspase-3 activation. Collectively, these findings suggest that TGM2 expression increases as a function of malignancy grade and tumor recurrence and that inhibition of TGM2 reduces meningioma cell growth.

## Introduction

Meningiomas are the second common primary tumor of the central nervous system (CNS), accounting for 20 to 27% of CNS tumors [1]. They arise from arachnoid cells of the leptomeninges in the brain and spinal cord, and a recent study identified a prostaglandin D synthase-positive meningeal precursor as the cell of origin for meningioma [2]. The histology of the tumors are diverse, and exhibit highly variable clinical characteristics [3]. Most meningiomas are benign, while some tumors are prone to recurrence and cannot be treated without causing major neurological deficits or even lethality.

Compared to other malignant brain tumors, there is relatively little known about the molecular pathogenesis of meningioma or the specific molecular changes that may have prognostic value or represent targets for therapeutic drug design. *NF2* gene deletion and exposure to ionizing radiation are established risk factors, while the role of sex hormones or head injury has been proposed, but not proven. Substantial evidence from previous studies indicated that the main genetic event in meningioma initiation is inactivation of the *NF2* gene, accounting for 30 to 70% of sporadic meningiomas [4]. A recent genomic study in paired progressive meningiomas also demonstrated that *NF2* gene inactivation is an early and frequent event in progressing meningioma samples and is associated with higher chromosome instability during progression [5]. Moreover, animal studies showed that *Nf2* loss in arachnoid cells is rate-limiting for meningioma formation, additional inactivation of p16Ink4a increases the frequency of meningioma [6]. The mice with both *Nf2* and *Cdkn2ab* inactivation lead to short latency of tumor development and the ability to induce grade II/III meningioma progression [7]. Furthermore, losses on chromosomes 1p, 9p, 10q, and 14q as well as chromosomal gains on 12q, 17p, 17q, and 20q have been implicated in the malignant progression of meningiomas [8]. Some studies also showed that genes such as *TGF-β, VEGF-A, hTERT, MMP2, MMP9* or *TIMP-1* may also be related to meningioma progression [9].

Recently, several studies using cDNA microarray analysis have been reported and tried to unveil the association between gene factors and tumor aggressiveness and to discover the biomarkers for meningiomas [10]. However, the microarray results could vary considerably due to different sample sources. The expression pattern of meningiomas could be altered by intermixed with normal tissue in surgical specimen or cell line with multiple passages. Thus, in this study, we generated primary cultures of human meningiomas to avoid contamination from other cells in the specimen. The microarray analysis and immunohistochemical staining revealed that transglutaminase 2 (TGM2) expression was increased in higher-grade meningiomas and in recurrent tumors. We also demonstrated that inhibition of TGM2 reduces meningioma cell growth.

## Materials and Methods

### Ethics Statement

The study was approved by the Institutional Review Board of Chang Gung Memorial Hospital (IRB 98-3933B and 101-4601B). A written informed consent was obtained from all participants.

### Sample collection

Meningioma samples were acquired from surgical specimens. Fresh tissues were harvested for primary cultures as described below and/or were formalin-fixed for histological and immunohistochemical analyses. Clinical information was collected, including demographic data, tumor location, treatment options and prognosis.

### Cell cultures

Fresh tumor tissues were immersed in culture medium (Dulbecco's modified Eagle medium (DMEM), Gibco), minced, and digested with trypsin/EDTA. The cells were pelleted for 5 min at 1000 RPMs, re-suspended in DMEM with 10% fetal calf serum, seeded evenly into 6-well plates, and incubated at 37°C in a humidified atmosphere (5% CO_2_). Confluent cultures were split using 0.05% trypsin/EDTA, and medium was changed twice a week. The mRNA extractions and *in vitro* assays were performed before passage 5, to avoid loss of original phenotype from long-term passages.

### Microarray analysis

RNA was prepared from primary cultures using an RNA extraction kit (Geneaid, Taipei, Taiwan) following the standard protocol. RNA quality and integrity were verified (RNA integrity number >7). Expression profiling was carried out using Affymetrix U133 Plus 2.0 arrays (Affymetrix, Santa Clara, CA), which contain over 47,000 transcripts and variants, including 38,500 well-characterized human genes. A total of 24 samples were assayed, including 3 samples obtained from arachnoid membranes (Ara), 18 samples obtained from benign meningiomas (WHO grade I), and 3 samples obtained from atypical meningiomas (WHO grade II). Standard cDNA synthesis, probe labeling, hybridization, and scanning of the arrays were performed using the standard protocols in the Genomic Medicine Research Core Laboratory (GMRCL) of Chang Gung Memorial Hospital as previously described [11]. Basic microarray data visualization and data filtering were accomplished as previously described [12], [13].

**Figure 1:**
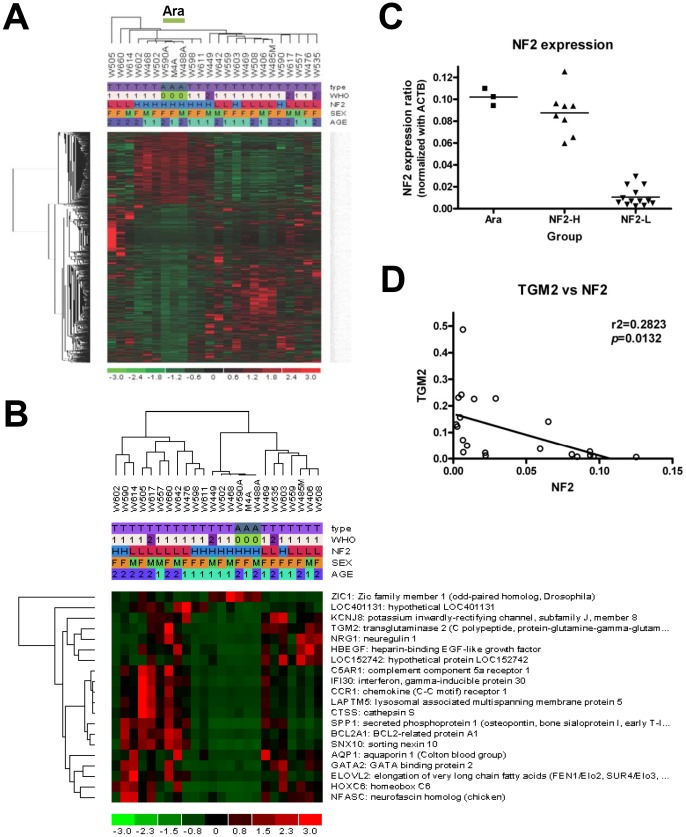
Microarray analysis of human meningiomas. (A) Expression profiles of 24 samples, including 3 samples from arachnoid membranes (green; Ara, WHO = 0), 18 samples from benign meningiomas (pink; grade I, WHO = 1), and 3 samples from atypical meningiomas (purple; grade II, WHO = 2). The heat map generated by hierarchical clustering revealed that the samples from arachnoid membranes clustered together. (type: A =  Arachnoid membrane, T =  Meningioma; WHO: WHO grades; NF2: H =  normal expression, L =  low expression; SEX: M =  male, F =  female; AGE: 1<60, 2≧60 y/o) (B) Comparison between arachnoid membranes and meningiomas identified 20 genes with significantly differential expression (fold change >4; corrected p (FDR) <0.05). (C) *NF2* gene expression levels were different, with low expression in 13 meningiomas. (Group: Ara, Arachnoid membrane; NF2-H and NF2-L, meningiomas with normal and low *NF2* expression, respectively) (D) TGM2 expression was inversely correlated with *NF2* gene expression. (Pearson correlation coefficient r = −0.5313, r2 = 0.2823, p = 0.0132).

The dCHIP program (build date-Dec 5, 2011) was used to analyze the array data by comparing each group and performing unsupervised tree-view clustering for all 24 array samples to produce a dendrogram [14]. Sample groupings were established, and invariant sets were normalized using median probe intensity as the baseline array parameter. Model-based expression was calculated using the Perfect Match/Mismatch difference model method. Initially, multivariate analysis was performed using the Compare Samples tool in which the mean Experimental/Base (Tumor/Ara) expression ratio was greater than 1.2, the *p*-value for a paired t-test was less than 0.05, and the samples were permuted 50 times to assess the false discovery rate. Filtered gene lists consisting of 2547 transcripts were clustered by sample and gene using the default parameters. The heat map generated by hierarchical clustering revealed that the samples from arachnoid membranes clustered together and fell into the group of tumors with high NF2 expression (Figure 1).

### Immunohistochemistry (IHC)

Tissue specimens or cultured cells were fixed with 4% paraformaldehyde in phosphate-buffered saline (PBS) for IHC analyses. Paraffin-embedded tissue sections from a different cohort (n = 82, WHO grade I = 58, grade II = 21, and grade III = 3) were immunostained using previously described conditions. Briefly, the sections were de-paraffinized using xylene, rehydrated and blocked with 2% goat serum. Samples were subsequently incubated with primary antibody overnight at 4°C and secondary antibody conjugated to Horseradish Peroxidase (HRP) at room temperature for 1 hour. The signals were visualized with an HRP substrate. The following primary antibodies were used: polyclonal rabbit anti-GFAP (glial fibrillary astrocytic protein) (Chemicon International, Temecula, CA), monoclonal mouse anti-EMA (epithelial membrane antigen) (Thermo Scientific, Rockford, IL), polyclonal rabbit anti-S100 and monoclonal mouse anti-vimentin (Dako, Denmark) antibodies. Transglutaminase 2 (TGM2) antibody was purchased from Abcam (Cambridge, UK). At least 4 high-power fields (HPF, 400x) were randomly photographed from the tumor region. In each HPF, individual cell counts were made by single observer and mean percentage were recorded as %/HPF.

### Immunoblot analysis

Meningioma cells were harvested by washing cell layers twice in cold PBS and then lysed with RIPA buffer (50 mM Tris, pH 8.0, 150 mM NaCl, 1% Nonidet P-40, 0.5% deoxycholate acid, 0.1% SDS and 2 mM EDTA) containing phosphatase inhibitors (2 mM sodium orthovanadate, 50 mM sodium fluoride and 50 nM calyculin A) and a 1× complete protease inhibitor cocktail (Roche Molecular Biochemicals, Indianapolis, IN). The cell lysates were incubated for 30 min on ice, clarified by centrifugation (13,000×g) and re-suspended in 1× sample buffer (33% glycerol, 6.7% SDS, 330 mM dithiothreitol). Proteins were resolved by SDS-PAGE, transferred to nitrocellulose membranes (Bio-Rad, Hercules, CA) and subjected to immunoblot analysis using the appropriate antibodies, HRP-conjugated secondary antibodies and the enhanced chemiluminescence (ECL) detection systems (GE, Fairfield, CT). Relative expression levels were quantified on a GS-800 Calibrated Densitometer (BioRad), and *p*-values were determined using a paired Student's t-test. Antibodies for cleaved and total caspase-3, phospho- and total AKT, and α-tubulin were purchased from Santa Cruz Biotechnology and Cell Signaling Technology.

### Cell viability assay

Cell viability was tested by a 3-(4,5dimethylthiazol-2-yl)-2,5-diphenyltetrazolium bromide (MTT)-based survival assay (Sigma-Aldrich, St. Louis, MO. USA) as published previously. Briefly, MTT (5 mg/ml) was added to the medium in 96-well plates containing 2500 meningioma cells; after 4 hours, the medium was removed and DMSO was added. The optical density at 540 nm was detected by an ELISA reader (Infinite M200 Pro, Tecan, Switzerland).

### siRNA transfection

For *in vitro* gene knockdown experiments, siRNAs (FlexiTube siRNA: Hs_TGM2_1, 2, 5, 6, 7) were purchased from Qiagen (Venlo, Netherlands). The transfection was performed according to the standard protocol from manufacturer [15]. Briefly, culture medium was replaced with serum-free medium before mixture with the diluted siRNAs along with lipofectamin 2000 (Invitrogen, Calsbad, CA, USA) for 4 hours; then change medium with 10% serum and extracted RNA/protein after 24–72 hours as described previously. The knockdown efficiency was calculated using real-time quantitative PCR using specific primers and SYBR Green I Master reagent with Lightcycler 480 (Roche Diagnostics, Mannheim, Germany).

### Statistical analyses

Standard statistical analyses were used to test the difference among groups. All data were expressed as mean ± SD and a p value lower than.05 was considered significant. Data were analyzed by using commercially available statistical software SPSS (SPSS Inc, Chicago, IL) and GraphPad Prism (GraphPad Software Inc. La Jolla, CA). All in vitro experiments were performed at least three times with similar results.

## Results

### Human meningioma cultures

For this study, we established primary meningioma cultures comprised of epithelial-like cells expressing vimentin [16], epithelial membrane antigen (EMA) [17], and S100β [18], but not glial fibrillary astrocytic protein (GFAP) (Figure S1 in File S1). All mRNA extractions and *in vitro* assays were performed before passage 5 to avoid loss of the original phenotype from long-term passage.

### Expression profiling

The 24 samples used for microarray analysis included 3 from arachnoid membranes (green; Ara, WHO = 0), 18 from benign meningiomas (pink; grade I, WHO = 1), and 3 from atypical meningiomas (purple; grade II, WHO = 2). The clinical information is listed in Table S1 in File S1. The heat map generated by hierarchical clustering revealed a differential expression pattern of 2547 transcripts in 24 samples after multivariate analysis (Figure 1A). Interestingly, samples from arachnoid membrane and those with *NF2* gene expression clustered together. Further analysis was performed with MATLAB version (R2013a) using the “mafdr” command to calculate the estimate false discovery rate (FDR) for multiple hypothesis testing of comparison between arachnoid membranes and meningiomas. The 20 genes with significantly differential expression were listed in Table 1 and the heat map was generated by hierarchical clustering (Figure 1B). Compared with the arachnoid membranes, *TGM2* gene expression was 11.33-fold higher in meningiomas (*p* = 0.001429; FDR *p* = 0.034055289). Besides, *NF2* gene expression was low in 13 of the 21 meningiomas analyzed (Figure 1C). *TGM2* gene expression was inversely correlated with *NF2* gene expression (r2 = 0.2823, p = 0.0132) (Figure 1D).

**Table 1:** Fold change and statistical significance for genes differentially expressed between Arachnoid membranes and Meningiomas.

Probeset ID	Gene Symbol	Fold Change	*P*	*P* (FDR)*
236896_at	*ZIC1*	−3.37	0.025576	0.049611751
206343_s_at	*NRG1*	5.79	0.002985	0.030487365
205303_at	*KCNJ8*	6.55	0.00062	0.031028682
213438_at	*NFASC*	9.88	0.000319	0.031929514
205681_at	*BCL2A1*	7.63	0.001923	0.029163321
209047_at	*AQP1*	8.36	0.002824	0.030724052
237737_at	*LOC401131*	8.51	0.000658	0.027442033
209710_at	*GATA2*	8.14	0.002164	0.030083364
201422_at	*IFI30*	7.73	0.001366	0.040213643
203821_at	*HBEGF*	7.73	0.000549	0.030528219
202901_x_at	*CTSS*	8.15	0.004221	0.034630372
201042_at	*TGM2*	11.33	0.001429	0.034055289
213712_at	*ELOVL2*	16.62	0.004042	0.034877067
206858_s_at	*HOXC6*	18.38	0.000098	0.016348445
240167_at	*LOC152742*	18.89	0.001614	0.032309866
218404_at	*SNX10*	18.32	0.000094	0.023521743
220088_at	*C5AR1*	35.59	0.002852	0.030368497
205098_at	*CCR1*	38.14	0.005182	0.036019406
201721_s_at	*LAPTM5*	60.22	0.004092	0.034710051
209875_s_at	*SPP1*	160.42	0.00009	0.045041635

*The estimate false discovery rate (FDR) for multiple hypothesis testing were performed with MATLAB version (R2013a) using the “mafdr” command.

### TGM2 expression in meningiomas

**Figure 2:**
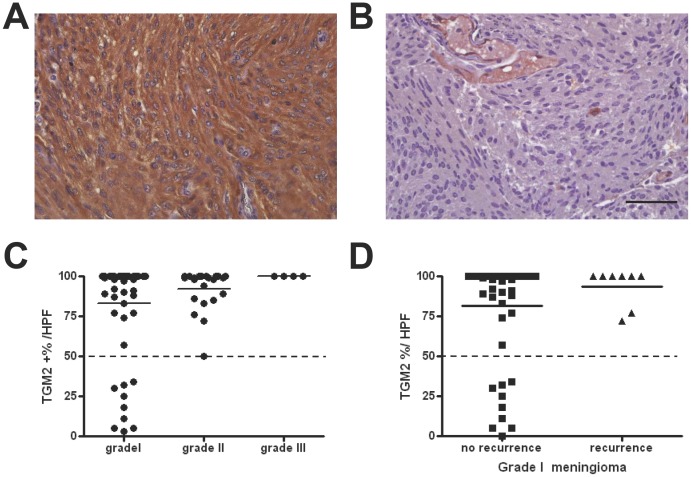
Validation of TGM2 by immunohistochemical study. TGM2 immunohistochemistry (IHC) was performed on 82 meningiomas from a different cohort (n = 82, WHO grade I = 58, grade II = 21, and grade III = 3). The IHC images represented high (A) and low (B) expression of TGM2, respectively. Scale bar  = 50 µm. (C) The quantification of TGM2 expression was expressed by percentage of TGM2-immunopositive cell per high power field (%HPF). The dot plot showed that TGM2 expression was abundant (positive in >50% of cells) in all grade II and III meningiomas. The TGM2 expression level was correlated with increased WHO malignancy grade (ANOVA, p = 0.0046). (D) The dot blot for TGM2 expression in grade I meningiomas revealed that those tumors with recurrence had high TGM2 expression (positive in >70% of cells).

From the microarray analysis, we choose TGM2 for further study, because TGM2 has been reported as a prognostic marker for laryngeal cancer [19] or colorectal cancer [20] as well as a chemotherapy-resistance marker for breast cancer [21] and lung cancer [22]. The inhibition of TGM2 may impair cell proliferation and induced apoptosis in gliomas [23], [24]. To validate the significance of differential expression of TGM2, a total of 82 meningioma samples were immunostained with a TGM2 antibody and quantified by microscopy under a 400X high power field (HPF) system (illustrated in Figure 2A and 2B). Abundant TGM2 expression (defined by >50% TGM2 immunopositive cells) was detected in all grade II and III meningiomas (Figure 2C). The expression level was correlated well with the WHO grading (ANOVA, p = 0.0046). For grade I meningioma, high TGM2 expression (>50%) was observed in those with recurrence, while benign tumors had low TGM2 expression, suggesting that TGM2 expression may induce more aggressive tumor behavior (Figure 2D).

### TGM2 inhibition induces cell death

**Figure 3:**
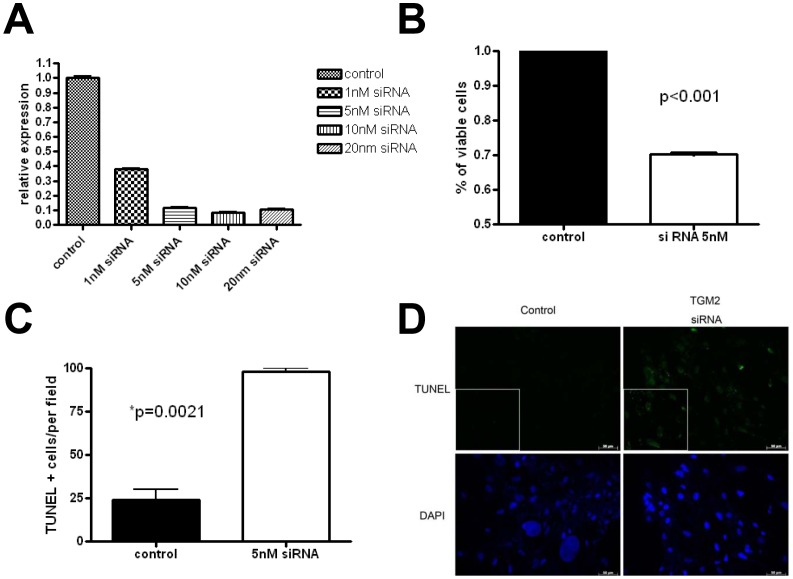
TGM2 inhibition by siRNA treatment induces cell apoptosis. (A) Quantitative RT-PCR using SYBR Green was used to assay *TGM2* mRNA expression in siRNA-treated cells. Meningioma cells treated with *TGM2* siRNA exhibited reduced TGM2 expression in a dose-dependent manner (>80% in 5 nM siRNA). (B) Cell viability assessed using the MTT assay showed 30% decrease of cell survival following 5 nM siRNA exposure. (C) and (D) TUNEL assays were used to measure apoptosis following 5 nM siRNA treatment. Quantification expressed by the number of TUNEL-positive per high power field, demonstrated that *TGM2* siRNA treatment increased meningioma cell apoptosis (unpaired Student's t-test, p = 0.0021).

Since the TGM2 expression was high in meningiomas and associated with the aggressive tumor behavior, we reasoned that TGM2 inhibition might be a therapeutic target for meningioma. For specific inhibition of TGM2 gene expression, we apply FlexiTube GeneSolution GS7052 for TGM2 from Qiagen, which contains Hs_TGM2_1, 5, 6, 7 clones [15]. The mixture of siRNA yielded an efficient knockdown of TGM2 expression in a dose dependent manner (Figure 3A). Treatment of meningioma cells with 5 nM siRNA decreased *TGM2* mRNA expression by 80–90% 2 days after transfection by quantitative RT-PCR and resulted in a 30% reduction in cell survival (Figure 3B). Furthermore, TGM2 inhibition by siRNA induced meningioma cell apoptosis (TUNEL + cells; Figure 3C and 3D).

**Figure 4:**
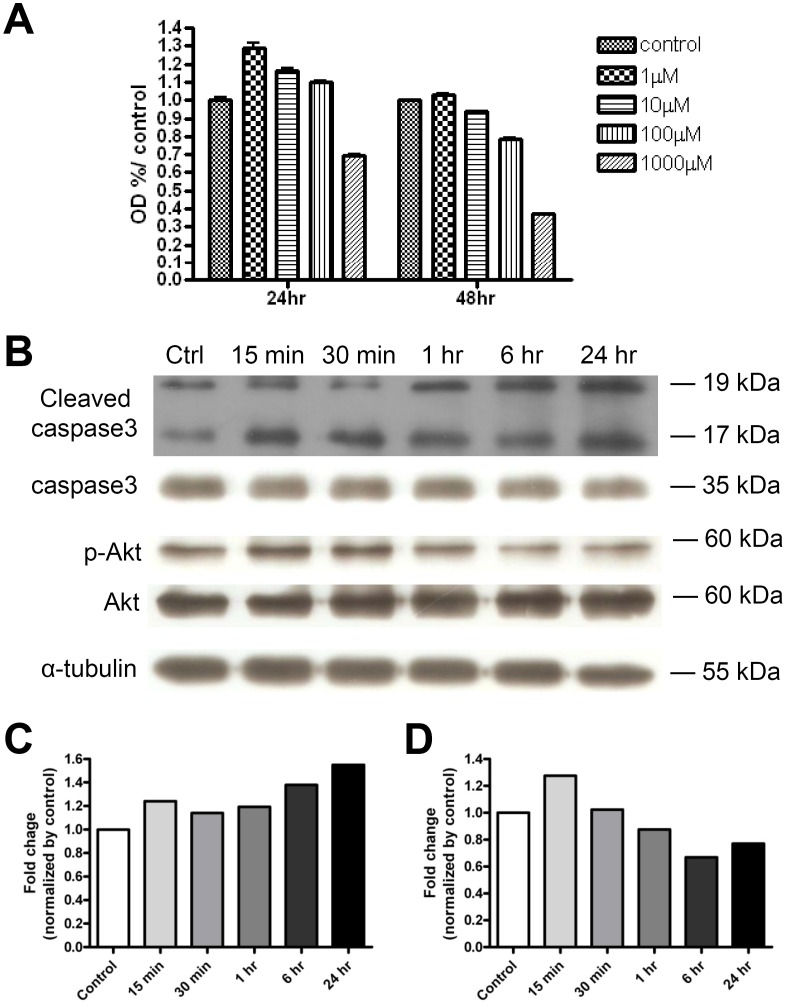
The molecular mechanism of reduction of meningioma cell survival by TGM2 inhibitor. (A) Cell viability was assessed by using the MTT assay and was expressed as percentage of control. Meningioma cell survival was measured at 24- and 48-hour after application of cystamine in 1, 10, 100, 1000 µM concentration. Meningioma cell viability was reduced to 39.6% of control 48 hours after cystamine treatment. (B) Western blotting for cleaved caspase-3 and p-Akt. Quantification of immunoblotting assay showed that cystamine resulted in (C) a 1.5-fold increase in caspase-3 activation relative to controls and (D) a 30% reduction in Akt phosphorylation (quantification of data from 4 independent experiments).

We next tested the effect of cystamine in meningioma cells. Cystamine is an orally active competitor of transglutaminase, which blocks access to the active site of the enzyme. TGM2 inhibitors have been reported as a treatment modality for various conditions, such as celiac sprue, Huntington's disease, and certain types of cancer [25]. Treatment of meningioma cells decreased survival in a dose-dependent fashion (Figure 4A). Meningioma cell viability reduced to a maximum of 39.6% of control after 48-hour of cystamine treatment.

To determine the molecular mechanism of cell death induced by genetic or pharmacological inhibition of TGM2, we initially examined caspase-3 activation. Treatment of meningioma cells with cystamine induced caspase-3 activation by 1.5-fold (Figure 4B and 4C), which was associated with a reduction in activated (phosphorylated) AKT expression.

## Discussion

Meningiomas are unique tumors of central nervous system and are understudied in comparison with malignant gliomas. Employing high-throughput microarray analyses, genes and pathways associated with tumorigenesis, clinical progression, response to treatment, and environmental exposure, can be discovered, leading to the development of new biomarkers and therapeutic targets. A few microarray studies of meningiomas in recent years with different aims and approaches have identified several targets; however, few commonly de-regulated genes have been identified [10]. One possible reason for the results may be various sources for sample preparation, in which tissue heterogeneity of surgical specimen can confound the interpretation of gene expression data. The surgical specimens contain not only tumor cells but also tumor vasculature, inflammatory cells, circulating leukocytes, and fibroblasts, while tumor cells are the dominant component of the primary culture. Although a previous expression profiling study comparing the fresh frozen specimen with meningioma cultures in passage 5 or 10 revealed dozens of differentially-expressed genes [26], our study using primary meningioma cultures with passage 3 has the advantage of homogenous cell type and closer to original tumor properties. Consistent with our finding of TGM2 upregulation in aggressive meningiomas, the microarray dataset GSE32197 from public GEO (Gene Expression Ominbus database, NIH, USA; http://www.ncbi.nlm.nih.gov/geo/) also revealed an 8-fold increase in *TGM2* expression in anaplastic meningioma relative to low-grade fibroblastic meningioma (Figure S2 in File S1) [27].

Transglutaminase 2 is a multi-functional enzyme that post-translationally modifies proteins by catalyzing the formation of intermolecular isopeptide bonds between glutamine and lysine side-chains. It plays a role in diverse biological functions, and is believed to participate in the pathogenesis of several unrelated diseases, including celiac sprue, neurodegenerative diseases, and certain types of cancer [28]. Recent studies showed that upregulation of TGM2 was associated with poor prognosis in hepatocellular carcinoma [29], colorectal cancer [20], non-small cell lung cancer [30], or laryngeal cancer [19]. Moreover, it had been reported as a chemotherapy-resistance marker for breast cancer [21] or lung cancer [22]. In addition, we analyzed the data publicly available at the REMBRANDT database (REpository of Molecular BRAin Neoplasia DaTa, NCI, NIH, USA; https://wiki.nci.nih.gov/display/icrportals/REMBRANDT). The patient survival data mining demonstrated significant detrimental effect of TGM2 upregulation in glioma with a Log-rank p-value of 0.0068. Furthermore, TGM2 inhibitor such as cystamine, glucosamine, or KCC009 effectively promote cell death in glioma [31], breast cancer [32], or pancreatic cancer [33]. These findings and our results suggest that TGM2 upregulation is associated with aggressive behavior of various tumors and may be a therapeutic target for some kinds of cancers.

Transglutaminase 2 is abundantly expressed in many tissues and widely distributed in various parts of a cell, including the extracellular matrix, plasma membrane, cytosol, mitochondria, and nucleus. It exerts opposing effects on cell growth, differentiation and apoptosis via multiple activities, including transamidase, GTPase, cell adhesion, protein disulfide isomerase, kinase and scaffold activities. The exact role of TGM2 in tumor formation is yet to be elucidated but several mechanisms are proposed. TGM2 can activate NF-κB and focal adhesion kinase (FAK) tyrosine kinase, in turn activate anti-apoptotic pathways to allow cancer cells to become immortalized [25]. In agreement with our findings of caspase-3 activation and reduced phosphorylation of Akt, a previous study showed that the TGM2 inhibitor KCC009 decreased Akt phosphorylation and upregulated the expression of the pro-apoptotic protein Bim, resulting in enhanced cell apoptosis in cultured mouse glioblastoma cell line [34].

In the present study, upregulation of TGM2 gene expression in meningiomas was identified by expression profiling, analysis of primary meningioma cultures, and immunohistochemical analyses using a distinct set of meningiomas all support the idea that TGM2 expression increases in a malignancy grade-dependent manner. We also demonstrated that inhibition of TGM2 decreased meningioma cell growth. Future studies focused on this interesting enzyme may lead to new treatment options for patients with this common CNS malignancy.

## Supporting Information

File S1 **File contains Table S1 and Figures S1 and S2.**
(DOC)
