# Supplementary material for: Transglutaminase 2 Expression Is Increased as a Function of Malignancy Grade and Negatively Regulates Cell Growth in Meningioma
Source: PLoS One. 2014 Sep 23;9(9):e108228. doi: 10.1371/journal.pone.0108228 (PMC4172767; doi:10.1371/journal.pone.0108228)
Supplement: File S1 — File contains Table S1 and Figures S1 and S2. (DOC) [file pone.0108228.s001.doc]

**Supporting information**

**Title: Transglutaminase 2 expression is increased as a function of malignancy grade and negatively regulates cell growth in meningioma**

Yin-Cheng Huang, M.D., Ph.D.1,5, Kuo-Chen Wei, M.D. 1,5, Chen-Nen Chang, .M.D., Ph.D. 1,5, Pin-Yuan Chen, M.D.,Ph.D. 1,5, Peng-Wei Hsu, M.D.1,5, Carl P. Chen, M.D., Ph.D. 4,5 , Chin-Song Lu, M.D.2,3,5, Hung-Li Wang, Ph.D.5, David H. Gutmann, M.D., Ph.D.6, Tu-Hsueh Yeh, M.D., Ph.D.2,3,5 *

^1^ Department of Neurosurgery, Chang Gung Memorial Hospital at Linkou, Taoyuan, Taiwan

^2^ Department of Neurology, Chang Gung Memorial Hospital at Linkou, Taoyuan, Taiwan;

^3^ Neuroscience Research Center, Chang Gung Memorial Hospital at Linkou, Taoyuan, Taiwan

^4^ Department of Rehabilitation, Chang Gung Memorial Hospital at Linkou, Taoyuan, Taiwan

^5^ Chang Gung University, College of Medicine, Taoyuan, Taiwan

^6^ Department of Neurology, Washington University, School of Medicine, St. Louis, MO, U.S.A.

**Table S1. Clinical information of the 24 samples for microarray analysis**

| **Sample** | **Sex** | **Age (years)** | **Type** | **WHO grading** |
| --- | --- | --- | --- | --- |
| M4A | F | 28 | A | 0 |
| W406 | M | 43 | T | 1 |
| W449 | M | 60 | T | 2 |
| W468 | M | 60 | T | 1 |
| W476 | F | 32 | T | 1 |
| W469 | F | 50 | T | 1 |
| W485M | F | 63 | T | 1 |
| W488A | M | 62 | A | 0 |
| W502 | F | 58 | T | 1 |
| W505 | F | 81 | T | 1 |
| W508 | F | 62 | T | 1 |
| W535 | F | 76 | T | 2 |
| W557 | M | 57 | T | 1 |
| W559 | F | 58 | T | 1 |
| W590 | F | 62 | T | 1 |
| W590A | F | 62 | A | 0 |
| W598 | F | 53 | T | 1 |
| W602 | F | 61 | T | 1 |
| W603 | F | 39 | T | 1 |
| W611 | F | 56 | T | 1 |
| W614 | M | 80 | T | 1 |
| W617 | M | 64 | T | 2 |
| W642 | M | 62 | T | 1 |
| W660 | F | 72 | T | 1 |

F: Female; M: Male; A: Arachnoid membrane; T: Meningioma;


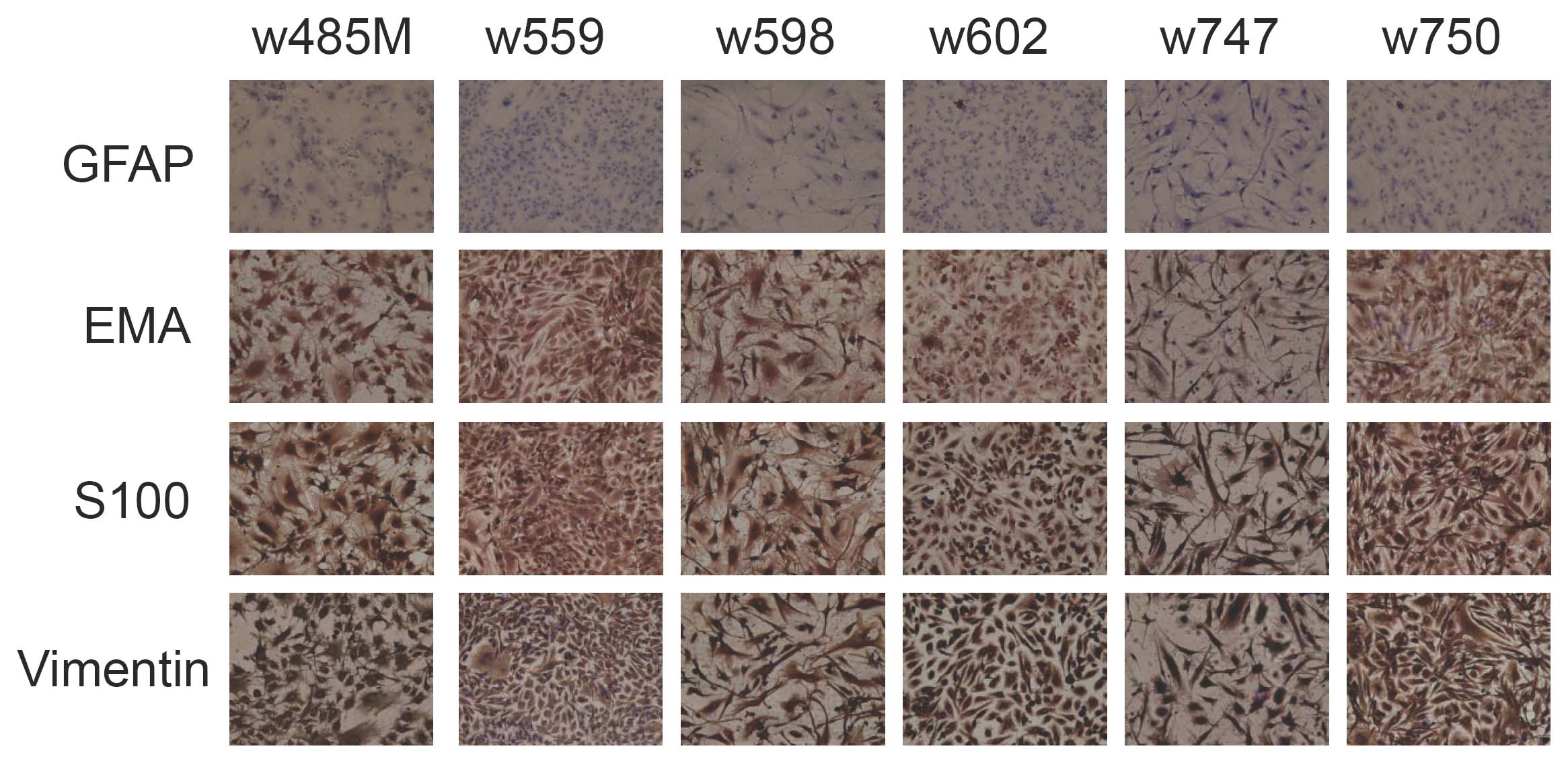


**Supplementary Figure S1. Characterization of meningioma cultures by immunohistochemistry.**

For each meningioma culture, GFAP, EMA, S100, and vimentin immunostaining was performed. Cells are positive for vimentin, EMA, S100β expression, but negative for GFAP staining.


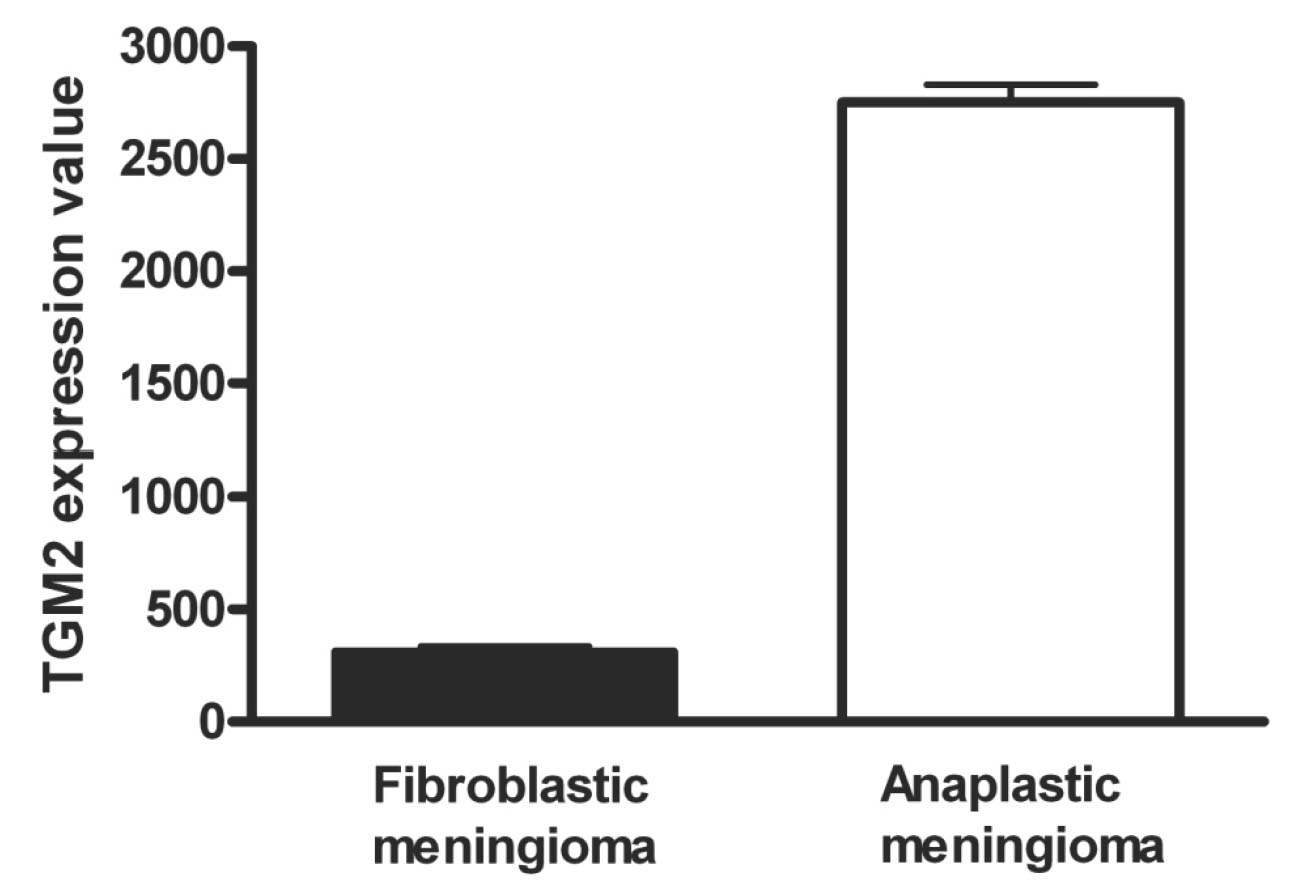


**Supplementary Figure S2. TGM2 expression was higher in anaplastic meningioma than fibroblastic meningioma.** The microarray dataset GSE32197 (Gene Expression Ominbus database, NIH, USA; <http://www.ncbi.nlm.nih.gov/geo/>) showed an 8-fold increase in *TGM2* expression in anaplastic meningioma relative to low grade fibroblastic meningioma.
